# The role of direct DNA repair gene O6-methylguanine-DNA methyltransferase (MGMT) in high grade malignant glioma

**DOI:** 10.1186/1755-8166-7-S1-P8

**Published:** 2014-01-21

**Authors:** M Jeru Manoj, G K Chetan, KVL Narasinga Rao, HN Venkatesh, MK Sibin, Ch Lavanya

**Affiliations:** 1 Department of Human Genetics NIMHANS, Bangalore-29, India; 2 Department of Neurosurgery, NIMHANS, Bangalore-29, India

## Background

High-grade [WHO, 2007- grade III-IV] gliomas are undifferentiated or anaplastic, are highly infiltrating and termed as malignant. The overall prognosis and survival rate is very low. Only few highly sensitive and specific biomarkers for Glioma have been identified, but are yet to be put for routine use due to challenges in their clinical validation for early disease detection, diagnosis and monitoring to improve long-term survival of patients. The inefficacy of currently available chemotherapeutic and radio-therapeutic agents largely depends on a number of resistance mechanisms among which DNA repair plays an important role. Hence bio-molecules involved in DNA repair mechanisms will be promising biomarkers. Current status in the validation of DNA repair genes include, studying the promoter methylation of the only direct DNA repair gene O6-methylguanine-DNA methyltransferase (MGMT). MGMT is epigenetically inactivated via hypermethylation of the 5′-CpG islands located in promoter region. It is associated with prediction of successful alkylating agent therapy with temozolomide in combination with radiotherapy and also longer overall survival in patients. The silenced MGMT gene possibly enhances radiation effects by inducing radiation-mediated double stranded DNA (dsDNA) breaks and suppression of dsDNA repair pathways after radiation exposure.

## Materials & methods

Post surgery excised tumor samples from 20 patients diagnosed histopathologically with high grade glioma were tested for MGMT promoter region methylation status. DNA extraction and bisulphate conversion using suitable commercially available kits (Qiagen) followed by Methylation specific PCR using specific methylated and unmethylated primers was carried out. Correlations are underway with clinical profile and treatment regimen.

## Results

Hypermethylation of the promoter region was observed in about 60%, (12) samples. The other samples only demonstrated unmethylated regions.

## Conclusions

Assessment of promoter methylation of the MGMT gene helps in the therapeutic choice of the patients. It has gained importance not only as a prognostic but also as a predictive biomarker in molecular profiling of high grade gliomas.

